# Enhanced Antioxidative Capacity Transfer between Sow and Fetus via the Gut–Placenta Axis with Dietary Selenium Yeast and Glycerol Monolaurate Supplementation during Pregnancy

**DOI:** 10.3390/antiox13020141

**Published:** 2024-01-23

**Authors:** Jiawen Zhang, Jun Wang, Ziwei Ma, Zhichao Fu, Yueqi Zhao, Xiangfang Zeng, Gang Lin, Shihai Zhang, Wutai Guan, Fang Chen

**Affiliations:** 1 College of Animal Science and National Engineering Research Center for Pig Breeding Industry, South China Agricultural University, Guangzhou 510642, China; zjw18533332024@163.com (J.Z.); wangjun19992022@163.com (J.W.); maziwei58856@163.com (Z.M.); 2543872938@stu.scau.edu.cn (Z.F.); 2695192284@stu.scau.edu.cn (Y.Z.); zhangshihai@scau.edu.cn (S.Z.); wtguan@scau.edu.cn (W.G.); 2 State Key Laboratory of Animal Nutrition, Ministry of Agriculture Feed Industry Center, China Agricultural University, Beijing 100193, China; ziyangzxf@163.com; 3 Institute of Quality Standards and Testing Technology for Agricultural Products, Chinese Academy of Agricultural Sciences, Beijing 100081, China; 18839609517@163.com; 4 Guangdong Laboratory of Modern Agriculture in Lingnan, Guangzhou 510642, China

**Keywords:** selenium yeast, glycerol monolaurate, antioxidative capacity, placenta, pregnancy

## Abstract

This study aims to investigate the impact of dietary supplementation with selenium yeast (SeY) and glycerol monolaurate (GML) on the transfer of antioxidative capacity between the mother and fetus during pregnancy and its underlying mechanisms. A total of 160 sows with similar body weight and parity of 3–6 parity sows were randomly and uniformly allocated to four groups (*n* = 40) as follows: CON group, SeY group, GML group, and SG (SeY + GML) group. Animal feeding started from the 85th day of gestation and continued to the day of delivery. The supplementation of SeY and GML resulted in increased placental weight and reduced lipopolysaccharide (LPS) levels in sow plasma, placental tissues, and piglet plasma. Furthermore, the redox balance and inflammatory markers exhibited significant improvements in the plasma of sows fed with either SeY or GML, as well as in their offspring. Moreover, the addition of SeY and GML activated the Nrf2 signaling pathway, while downregulating the expression of pro-inflammatory genes and proteins associated with inflammatory pathways (MAPK and NF-κB). Vascular angiogenesis and nutrient transportation (amino acids, fatty acids, and glucose) were upregulated, whereas apoptosis signaling pathways within the placenta were downregulated with the supplementation of SeY and GML. The integrity of the intestinal and placental barriers significantly improved, as indicated by the increased expression of ZO-1, occludin, and claudin-1, along with reduced levels of DLA and DAO with dietary treatment. Moreover, supplementation of SeY and GML increased the abundance of Christensenellaceae_R-7_group, Clostridium_sensus_stricto_1, and Bacteroidota, while decreasing levels of gut microbiota metabolites LPS and trimethylamine N-oxide. Correlation analysis demonstrated a significant negative relationship between plasma LPS levels and placental weight, oxidative stress, and inflammation. In summary, dietary supplementation of SeY and GML enhanced the transfer of antioxidative capacity between maternal-fetal during pregnancy via gut–placenta axis through modulating sow microbiota composition.

## 1. Introduction

Pregnancy is a series of temporary complex events, precisely coordinated with dramatic anatomical, physiological, and metabolic changes in the maternal body to meet the fetal intrauterine nutrient and oxygen requirements [1]. This period is characterized by systemic oxidative stress and chronic inflammation driven by elevated levels of reactive oxygen species (ROS) resulting from the extensive metabolic demands of both the mother and fetus [2]. Aberrant redox status during pregnancy induced by various factors, such as environmental stressors, emotional stress, or related diseases, has been shown to be a critical contributor to adverse pregnancy outcomes [3,4]. Moreover, maternal redox disturbance during pregnancy is well established as being closely associated with fetal oxidative stress, with potential long-term impacts on postnatal development and adult health [5].

The placenta plays a pivotal role in transmitting antioxidative capacity between the maternal and fetal domains during pregnancy, acting as a bridge that connects and mediates stress responses [6,7]. A substantial body of evidence demonstrates a close link between placental dysfunction, maternal redox imbalance, and adverse pregnancy outcomes [8,9]. Furthermore, research has indicated that placental redox disorder disrupts various facets of placental development and function, affecting vascularization, structural integrity, and substance exchange between mother and fetus, leading to a detrimental fetal intrauterine environment [10]. Lipopolysaccharide (LPS), originating from gut Gram-negative bacteria in the intestines, can trigger an inflammatory response when it enters extraintestinal organs through the bloodstream [11]. Placental exposure to LPS has been well documented to induce inflammation, disrupt barrier function, impede nutrient transport, and, ultimately, lead to adverse pregnancy outcomes [12,13]. Given these findings, we hypothesized that gut microbiota and the LPS metabolite may play critical roles in mediating the transfer of antioxidative capacity between the mother and fetus via placenta during pregnancy. In addition, we considered the possibility of dietary interventions aimed at regulating this site to mitigate oxidative stress in both the mother and fetus.

Supplementing functional additives with antioxidant and anti-inflammatory properties has become a widely recognized and effective method to improve reproductive health in both humans and animals today [14]. Selenium (Se), an essential trace element, acts as a potent antioxidant by being integral to selenoproteins and supporting antioxidant enzyme function, collectively safeguarding cells and tissues from oxidative damage [15]. Glycerol monolaurate (GML) exhibits potent antimicrobial, antiviral, and anti-inflammatory properties, effectively enhancing animal resistance to infections, immunity, and overall health [16]. Research conducted in sows has revealed that both Se and GML could enhance the redox balance, resulting in improved pregnancy outcomes by mitigating risks, such as intrauterine growth restriction, abortion, and infections [17,18,19,20]. Accumulating evidence demonstrates that the gut microbiota composition undergoes modification and reshaping in animals supplemented with Se and GML, implying that the gut microbiota could potentially function as a beneficial mechanism for enhancing reproductive health [21,22]. But the current understanding is limited regarding whether these compounds facilitate the transfer of antioxidative capacity between maternal and fetal compartments to ensure fetal intrauterine redox stability, as well as the role of microbiota in this process.

In this study, high-fertility sows were selected as the reproductive animal model due to their more pronounced oxidative stress during pregnancy, carrying up to 15–17 fetuses [23], to investigate the effects and underlying mechanisms of Se and GML on the transfer of antioxidative capacity between the maternal and fetal compartments. Firstly, we confirmed that gut-derived LPS participates in mediating antioxidative capacity transfer by determining systemic LPS levels, barrier integrity, oxidative status, and inflammation response throughout the maternal–placental–fetal axis, as well as conducting a correlation analysis of LPS and stress indicators. In addition, placental development, angiogenesis, and nutrient transportation were determined to investigate in more depth the mechanisms underlying the transmission of antioxidative capacity. Furthermore, the microbiome, metabolites, and correlations of serum LPS with key bacteria were determined to confirm the crucial role of the sow gut microbiome in this modulation.

## 2. Materials and Methods

### 2.1. Animals, Diets, and Management

All animal experiments were conducted following the guidelines of the Animal Ethics Committee of South China Agricultural University and approved by the Animal Conservation and Use Committee of South China Agricultural University (Approval No: SYXK2019-0136-1, Guangzhou, China). This experiment was conducted at an experimental farm in Heyuan city, Guangdong province, China. One hundred and sixty sows with parity levels of 3–6 (land sows × large white sows) and similar body weights were randomly and uniformly assigned to the following four groups (40 sows in each group): CON group (basal diet), SeY group (basal diet + 0.2 mg/kg selenium yeast (SeY)), GML group (basal diet + 0.1% GML), and SG group (basal diet + 0.2 mg/kg SeY + 0.1% GML). The nutrient composition of the basal diets complied with the nutritional requirements for gestating sows, as defined by the National Research Council (NRC) (2012). No antibiotics were used throughout the experiment. The feed composition and nutrient content of the basal diets are detailed in Table S1.

Feeding starts on the 85th day of gestation and continues until farrowing. The daily care and regular vaccination program for all sows and piglets was in accordance with the pig farm’s regulations. In the later stages of gestation (from day 85 to day 107 of gestation), each sow was kept in a separate pen (2 m × 0.6 mm^2^) with unrestricted access to water and provided with two meals per day (6:30 and 14:00). Sows had a limited intake, with a total intake of 3.0–3.5 kg per sow per day. Seven days prior to giving birth, the sows were transferred to individual farrowing crates (2 m × 1.5 m), with parturitions being regularly monitored in all groups. The farrowing room was carefully regulated, with disruptions minimized as much as possible, and the internal temperature was maintained at approximately 20 to 25 °C by an air conditioning system.

### 2.2. Sample Collection

On the 110th day of gestation, 6 healthy sows exhibiting normal body conditions and showing no abnormal clinical symptoms were randomly selected from each group, and a heparinized vacuum blood collection tube was used to collect blood (10 mL) from the marginal ear vein. Plasma samples were then obtained by centrifuging the blood samples at 845× *g* at 4 °C for 15 min and storing at –80 °C until analysis. Meanwhile, the selected healthy sows were subjected to rectal massage stimulation to collect fresh feces, which were immediately placed and stored in a −80 °C refrigerator for subsequent analysis.

On the day of farrowing, newborn piglets of the 6 sows selected from each of the previous groups (one piglet per litter, chosen based on the body weight closest to the average body weight of the litter) were randomly selected for anterior vena cava blood (5 mL) collection before suckling. The placentas of the selected sows were collected and weighed together and recorded as a litter placenta weight. Then, fresh placental samples were collected from areas with numerous blood vessels, approximately 5 cm away from the center of the placenta (umbilical cord), an area we consider to be representative of the overall placental status. The samples were surgically obtained by extracting approximately 1.5 × 1.5 cm (100 g) of cut placental tissue. The collected tissue was then further cut into pieces to ensure their uniformity, and these pieces were subsequently subpackaged and stored in liquid nitrogen until analysis.

### 2.3. Se Content Measurement

Weighed 0.5 g of placenta sample and accurately transferred 0.3 mL of plasma sample into a microwave digestion inner tank (DigiPrep; SCP Science, Courtaboeuf, France). Added 10 mL of nitric acid and 5 mL of H_2_O_2_, covered overnight, and followed the standard operating procedures of the microwave digestion instrument for digestion. Placed the digestion tank on a temperature-controlled electric heating plate, heated it at 100 °C for 30 min, and brought it to a constant volume of 25 mL with water. Finally, the total selenium content was measured using inductively coupled plasma mass spectrometry (ICP-MS, Agilent 1260/7700X, Santa Clara, CA, USA).

### 2.4. Chemical Analyses

Total antioxidant capacity (T-AOC), superoxide dismutase (SOD), glutathione (GSH), glutathione peroxidase (GSH-Px), and malondialdehyde (MDA) were determined using kits supplied by Nanjing Jianjian Biological Research Institute (NJBRI) and according to the guidelines. Diamine oxidase (DAO), D-lactic acid (DLA), and lipopolysaccharide (LPS) were measured in the sow plasma samples, and interleukin-1β (IL-1β), interleukin-6 (IL-6), interleukin-8 (IL-8), interleukin-18 (IL-18), tumor necrosis factor (TNF-α), and LPS were measured in the placenta and newborn piglet plasma samples.

Weigh 100 mg of sow feces, add PBS (pH = 7.2–7.4; concentration = 0.01 mol/L), homogenize at a 1:9 ratio, and then centrifuge the supernatant at 7104 *g* for 25 min. Afterward, remove the supernatant for the LPS and trimethylamine oxide (TMAO) detection. Porcine ELISA Kits from ML Bio Company (Shanghai, China) were utilized for these analyses. A comprehensive list of all kits employed in this study is provided in Table S2.

### 2.5. Total RNA Extraction and Real-Time Quantitative PCR

Take 50 mg of porcine placental tissue and place it in a 2.5 mL nonenzymatic grinding tube; then, add 500 microliters of lysis buffer (EZB-RN001, EZBioscience, Roseville, MN, USA). Follow the instructions for the lysis, RNA binding to the spin column, washing, elution, and centrifugation steps to obtain RNA (EZB-RN001-plus). Measure the RNA absorbance ratio (A260/A280) of all RNA samples using a spectrophotometer. Prepare the cDNA according to the manufacturer’s instructions for the RNA reverse transcription reaction (A0010CGQ, EZBioscience). The reaction protocol includes an initial denaturation cycle at 95 °C for 2 min, followed by 40 amplification cycles at 95 °C for 15 s and annealing at 60 °C for 30 s. Use the relative quantification method (2^−ΔΔCt^) to determine the relative mRNA expression, with β-actin as the internal reference, to determine the relative expression level of the target gene. Refer to Supplementary Table S3 for the primers used in this study.

### 2.6. Western Blotting Analysis

Use a total of 100 mg of pig placental tissue to extract the total protein with 0.5 mL of RIPA lysis buffer (Beyotime, Shanghai, China) containing 1% phosphatase inhibitor and 1% PMSF protease inhibitor. Then, separate 25 μg of total protein from each sample on a 10% SDS-PAGE gel, transfer to a polyvinylidene difluoride membrane (Millipore, Bedford, MA, USA), and block with 5% skimmed milk powder for 2 h. Wash the membrane 4 times with TBST buffer and then incubate with the primary antibody at 4 °C for 12–15 h. Information about the antibodies used in this study can be found in Table S4. Subsequently, wash the membrane and then incubate with the corresponding secondary antibody dilution for 1.5 h at room temperature. After the antibody incubation, wash the membrane 5 times with TBST. Finally, capture the signals on these membranes using the ECL Plus chemiluminescence detection kit (Applygen Technologies Inc., Beijing, China) and a chemiluminescence imaging analysis system (Tanon, Shanghai, China). Lastly, analyze the grayscale values using image processing software (ImagePro Plus 6.0) and normalize the relative protein expression levels using β-actin.

### 2.7. Bacterial Data Analysis

Genomic DNA was extracted from fresh sow feces using the CTAB method. The concentration and purity of the DNA were assessed with 1% agarose gel. The primers 341F (5′-CCTAYGGGRBGCASCAG-3′) and 806R (5′-GGACTACNNGGGTATCTAAT-3′) were employed to amplify the V3-V4 variable regions. For the PCR reaction, 15 μL of Phusion^®^ High Fidelity PCR Master Mix (New England Biolabs, Beijing, China) was used. The concentrations of the 341F and 806R primers mentioned above were 2 μmol/L, and for the template DNA, it was approximately 10 ng. The resulting PCR products were purified using the Qiagen Gel Purification Kit (Qiagen, Düsseldorf, Germany).

The sequencing libraries were prepared, according to the manufacturer’s instructions, using the TruSeq^®^ DNA PCR-Free Sample Preparation Kit (Illumina, San Diego, CA, USA), with the index codes added. The library quality was assessed using a Qubit@ 2.0 fluorometer (Thermo Scientific, Waltham, MA, USA) and an Agilent Bioanalyzer 2100 system. Subsequently, the sequencing of the 250 bp end-to-end reads was conducted on an Illumina Nova Seq platform, followed by the splicing of the end-to-end reads for raw markers using FLASH software (34.0.0.175). Data filtering and noise reduction were performed using QIIME software (version 1.9.1) to obtain a list of the ASVs and their features. Species annotation was then performed on the ASVs to acquire species information for each ASV.

### 2.8. Statistical Analysis

All data for the parameters of the sow and piglet sera and placental tissues were analyzed using one-way analysis of variance (ANOVA) and the general linear model procedure, arranged as a 2 × 2 factorial in a randomized complete block design in IBM SPSS statistical software (IBM Version 25.0, Armonk, NY, USA). Tukey’s post hoc test was further employed to assess the differences among the various groups. The regression analysis also used the same IBM SPSS statistical software (IBM SPSS Statistics 25). *p* < 0.1 was considered a trend, *p* < 0.05 was considered a significant difference, and *p* < 0.01 highly significant. All data are shown as the mean ± standard error (SEM).

The diversity of microbial species was assessed using alpha diversity metrics, including Shannon, ACE, and Chao1. All alpha diversity indices were calculated using QIIME (Version 1.7.0) and visualized using R software (Version 2.15.3). A beta diversity analysis was employed to compare the species complexity among the samples, and a weighted UniFrac beta diversity analysis was conducted using QIIME software (Version 1.9.1). Principal coordinate analysis (PCoA) was utilized to obtain the principal coordinates and visualize the complex, multidimensional data. Linear discriminant analysis (LDA) was applied to identify the bacterial groups present in each group using LEfSe.

## 3. Results

### 3.1. Effect of Dietary Addition of SeY and GML on Placental Weight and LPS Levels in Sow, Placenta, and Piglet

**Figure 1:**
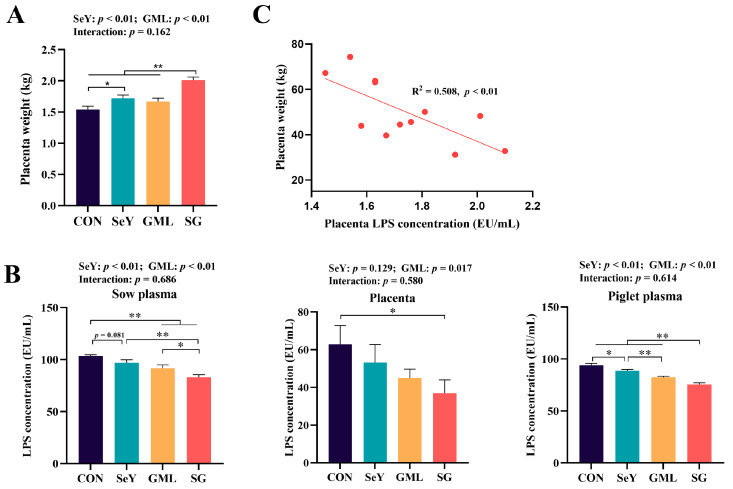
Litter placenta weight and sow plasma, sow placenta, and piglet plasma LPS levels: (**A**) maternal placenta weight; (**B**) sow plasm, sow placenta, and piglet plasma LPS levels; (**C**) regression analysis of placental weight and placental LPS content. CON, control; SeY, selenium yeast; GML, glycerol monolaurate; SG, selenium yeast + glycerol monolaurate. Data are presented as the means ± SEM; * *p* < 0.05 and ** *p* < 0.01.

To assess the placental development, as a key indicator of its function, we initially measured the placental weight in each group. We observed a significant increase in placental weight in sows fed with SeY (*p* < 0.01) (Figure 1A) and GML (*p* < 0.01) (Figure 1A). However, there was no significant difference between the SeY and GML groups, and no interaction between these two factors. Next, we analyzed the LPS levels in sow plasma, sow placenta, and piglet plasma. Our findings reveal that the addition dietary SeY significantly reduced sow plasma (*p* < 0.01) and piglet plasma (*p* < 0.01) LPS levels (Figure 1B). Similarly, dietary GML supplementation led to a significant decrease in sow plasma (*p* < 0.01), piglet plasma (*p* < 0.01), and placental (*p* < 0.05) LPS levels (Figure 1B). The correlation analysis found that the placental weight was significantly negatively correlated with placenta LPS level (R^2^ = 0.508, *p* < 0.01) (Figure 1C).

### 3.2. Effect of Dietary Addition of SeY and GML on Se Status along the Maternal–Placental–Fetal Axis

**Figure 2:**
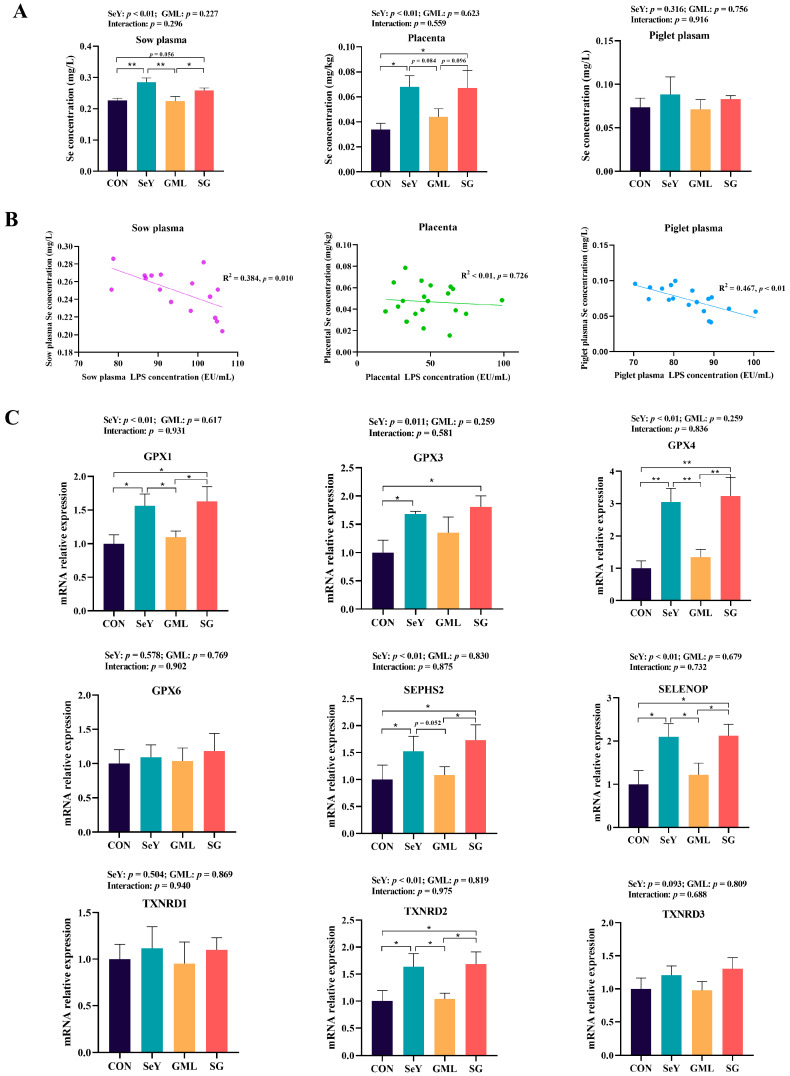
Transport of Se along the maternal–placental–fetal axis: (**A**) total Se content in the sow plasma, sow placenta, and piglet plasma; (**B**) regression analysis of the total Se content in sow plasma, sow placenta, and piglet plasma LPS content; (**C**) placental selenoprotein mRNA expression. CON, control; SeY, selenium yeast; GML, glycerol monolaurate; SG, selenium yeast + glycerol monolaurate. Data are presented as the means ± SEM; * *p* < 0.05 and ** *p* < 0.01.

Figure 2A shows that the dietary addition of SeY significantly increased the Se levels in the sow plasma (*p* < 0.01) and placenta (*p* < 0.01). We simultaneously performed a regression analysis to investigate the potential relationship between LPS and Se status (Figure 2B) and found that the Se level in the sow plasma was significantly negatively correlated with its LPS level (R^2^ = 0.384, *p* < 0.05), and the Se level in the piglet plasma was extremely significantly negatively correlated with its LPS level (R^2^ = 0.467, *p* < 0.01). The dietary supplementation of SeY (in the SeY and SG groups) significantly enhanced placental selenoproteins’ gene expression, including *GPX1* (*p* < 0.01), *GPX4* (*p* < 0.01), *SEPHS2* (*p* < 0.01), *SELENOP* (*p* < 0.01), *TXNRD2* (*p* < 0.01), and *GPX3* (*p* < 0.05) (Figure 2C). GML had no significant impact on both the Se levels and the gene expression of selenoproteins.

### 3.3. Effect of Dietary Addition of SeY and GML on Oxidative Stress in the Maternal–Placental–Fetal Axis 

**Figure 3:**
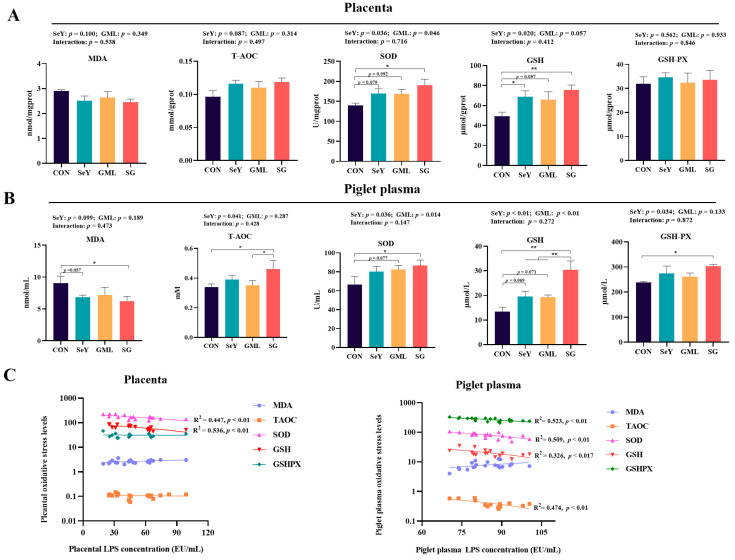
Maternal–placental–fetal axis redox status: (**A**) T-AOC, MDA, SOD, GSH, and GSH-PX activities in the placenta; (**B**) T-AOC, MDA, SOD, GSH, and GSH-PX activities in the piglet plasma; (**C**) regression analysis of the placental and piglet plasma redox statuses with the LPS content. CON, control; SeY, selenium yeast; GML, glycerol monolaurate; SG, selenium yeast + glycerol monolaurate. Data are presented as the means ± SEM; * *p* < 0.05 and ** *p* < 0.01.

The results pertaining to the redox status within the placental–fetal axis are depicted in Figure 3. The inclusion of dietary SeY led to a significant increase in placental SOD (*p* < 0.05) and GSH (*p* < 0.05) activities, with a noticeable trend toward elevated levels of T-AOC (*p* = 0.087) (Figure 3A). Furthermore, it resulted in heightened T-AOC (*p* < 0.05), SOD (*p* < 0.05), GSH (*p* < 0.01), and GSH-PX (*p* < 0.05) activities in piglet plasma, coupled with a tendency toward reduced MDA levels (*p* = 0.099) (Figure 3B). Similarly, dietary supplementation of GML significantly boosted placental SOD activity (*p* < 0.05) and exhibited a tendency toward increased GSH levels (*p* = 0.057) (Figure 3A) while also raising plasma SOD (*p* < 0.05) and GSH (*p* < 0.01) activities in piglets (Figure 3B).

Subsequently, we explored the relationship between these oxidative stress indicators and LPS. As illustrated in Figure 3C, the results demonstrate significant negative correlations between the antioxidant indicators SOD (R^2^ = 0.447, *p* < 0.01) and GSH (R^2^ = 0.536, *p* < 0.01) with placental LPS level. Moreover, piglet plasma antioxidant indicators including T-AOC (R^2^ = 0.474, *p* < 0.01), SOD (R^2^ = 0.509, *p* < 0.01), GSH (R^2^ = 0.326, *p* < 0.05), and GSH-PX (R^2^ = 0.523, *p* < 0.01), exhibited significant negative correlations with the LPS level in the piglet plasma.

**Figure 4:**
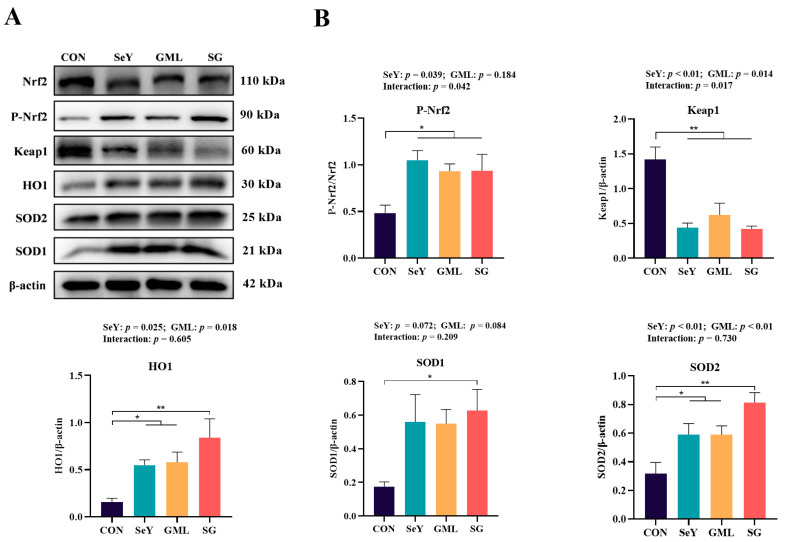
Maternal–placental–fetal axis Nrf2 antioxidant pathway: (**A**) Nrf2 antioxidant pathway Western blot results; (**B**) protein expression of placental Nrf2 antioxidant pathway (P-Nrf2/Nrf2, Keap1, SOD1, SOD2, and HO-1). CON, control; SeY, selenium yeast; GML, glycerol monolaurate; SG, selenium yeast + glycerol monolaurate. Data are presented as the means ± SEM; * *p* < 0.05 and ** *p* < 0.01.

To further elucidate the molecular mechanisms by which dietary SeY and GML enhance placental antioxidant capacity, we examined the expression of proteins associated with the Nrf2 pathway (Nrf2, Keap1, SOD1, SOD2, and HO-1), as depicted in Figure 4. The inclusion of SeY in the diet resulted in a significant increase in P-Nrf2 (*p* < 0.05), and HO-1 (*p* < 0.05), and a highly significant elevation in SOD2 (*p* < 0.01), while reducing the protein expression of Keap1 (*p* < 0.01). The inclusion of GML led to a remarkable decrease in placental Keap1 protein expression (*p* < 0.05) while significantly increasing the expression of SOD2 (*p* < 0.01) and HO-1 (*p* < 0.05). Notably, there was a significant interaction effect (*p* < 0.05) between SeY and GML for placental P-Nrf2 and Keap1 protein expression.

### 3.4. Effect of Dietary Addition of SeY and GML on Inflammation along the Placenta–Piglets Axis

**Figure 5:**
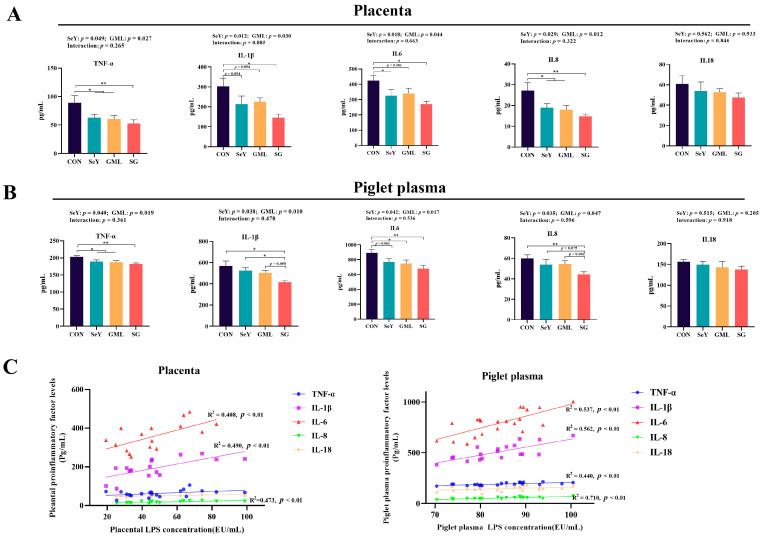
Inflammation levels in placenta and piglet plasma: (**A**) levels of pro-inflammatory factors TNF-α, IL-1β, IL-6, IL-8, and IL-18 in the placenta; (**B**) levels of pro-inflammatory factors (TNF-α, IL-1β, IL-6, IL-8, and IL-18) in the plasma of piglets; (**C**) regression analysis of plasma levels of pro-inflammatory factors in placenta, piglets, and their LPS content. CON, control; SeY, selenium yeast; GML, glycerol monolaurate; SG, selenium yeast + glycerol monolaurate. Data are presented as the means ± SEM; * *p* < 0.05 and ** *p* < 0.01.

**Figure 6:**
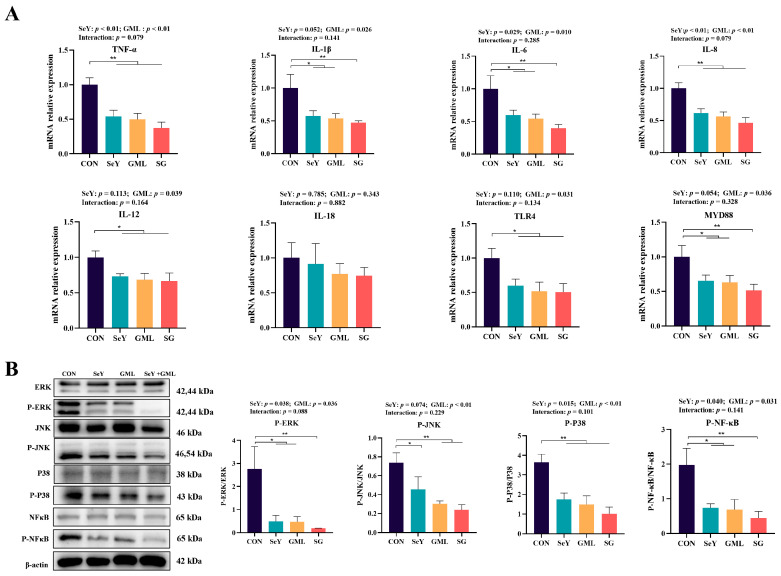
Expression of placental pro-inflammatory cytokine genes and inflammatory pathway protein expression: (**A**) mRNA expression of placental proinflammatory factors (*TNF-α*, *IL-1β*, *IL-6*, *IL-8*, *IL-12*, *IL-18*, *TLR4*, and *MYD88*); (**B**) expression of placental MAPK (P-ERK/ERK, P-JNK/JNK, and P-P8/P38) and NF-κB signaling pathway-related proteins. CON, control; SeY, selenium yeast; GML, glycerol monolaurate; SG, selenium yeast + glycerol monolaurate. Data are presented as the means ± SEM, * *p* < 0.05; ** *p* < 0.01.

Considering that redox imbalance often triggers inflammation, a major contributor to adverse pregnancy outcomes, we assessed several key markers associated with the inflammatory response in the placenta and piglets, as presented in Figure 5 and Figure 6. Our results demonstrate that the addition of SeY to the diet led to a significant reduction in placental TNF-α (*p* < 0.05), IL-1β (*p* < 0.05), IL-6 (*p* < 0.05), and IL-8 (*p* < 0.05) levels (Figure 5A). Furthermore, the piglet plasma levels of TNF-α (*p* < 0.05), IL-1β (*p* < 0.05), IL-6 (*p* < 0.05), and IL-8 (*p* < 0.05) also significantly decreased (Figure 5B) with SeY supplementation. Similarly, the addition of GML to the diet resulted in a significant decrease in placental TNF-α (*p* < 0.05), IL-1β (*p* < 0.05), IL-6 (*p* < 0.05), and IL-8 (*p* < 0.05) levels (Figure 5A). Piglet plasma levels of TNF-α (*p* < 0.05), IL-1β (*p* < 0.05), IL-6 (*p* < 0.05), and IL-8 (*p* < 0.05) were also significantly reduced (Figure 5B).

Then, we analyzed the relationship between the pro-inflammatory factors and LPS. The results show that the placental IL-1β (R^2^ = 0.490, *p* < 0.01), IL-6 (R^2^ = 0.408, *p* < 0.01), and IL-8 (R^2^ = 0.473, *p* < 0.01) levels were extremely significantly positively correlated with placental LPS content; piglet plasma TNF-α (R^2^ = 0.440, *p* < 0.01), IL-1β (R^2^ = 0.562, *p* < 0.01), IL-6 (R^2^ = 0.537, *p* < 0.01), and IL-8 (R^2^ = 0.710, *p* < 0.01) levels were exceedingly significantly positively correlated with piglet plasma LPS contents (Figure 5C).

We further examined pro-inflammatory-related genes (Figure 6A) and the activation of the MAPK and NF-κB signaling pathways (Figure 6B), which are crucial signaling pathways regulating inflammation in the placenta. The findings reveal that supplementing SeY resulted in a highly significant reduction in placental *TNF-α* (*p* < 0.01) and *IL-8* (*p* < 0.01) gene expression, accompanied by a significant decrease in *IL-6* (*p* < 0.05). Additionally, there was a noticeable trend toward the decreased expression of *IL-1β* (*p* = 0.052) and *MYD88* (*p* = 0.074). The introduction of GML into the diet also led to a remarkable decline in placental *TNF-α* (*p* < 0.01) and *IL-8* (*p* < 0.01) gene expressions, while significantly reducing the expression of *IL-1β* (*p* < 0.05), *IL-6* (*p* < 0.05), *IL-12* (*p* < 0.05), *TLR4* (*p* < 0.05), and *MYD88* (*p* < 0.05) genes. The addition of SeY to the diet significantly decreased the expression of P-ERK (*p* < 0.05), P-P38 (*p* < 0.05), and P-NF-κB (*p* < 0.05) proteins, with a noticeable trend toward reduced expression of P-JNK protein (*p* = 0.074). Similarly, incorporating GML into the diet significantly decreased the expression of P-ERK (*p* < 0.05) and P-NF-κB (*p* < 0.05) proteins, while also causing a highly significant reduction in the expression of P-JNK (*p* < 0.01) and P-P38 (*p* < 0.01) proteins.

### 3.5. Effect of Dietary Addition of SeY and GML on the Intestinal and Placental Barriers in Sows

**Figure 7:**
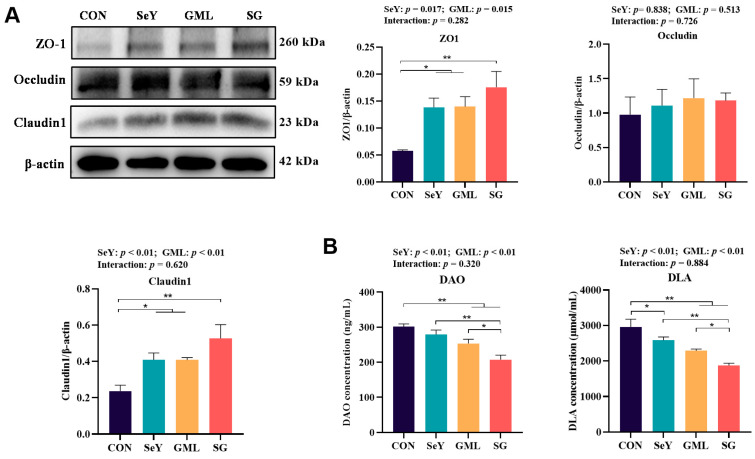
Maternal gut-placental barrier: (**A**) placental tight junction (ZO-1, Occludin, and Claudin-1) protein expression; (**B**) maternal intestinal permeability index (DAO and DLA). CON, control; SeY, selenium yeast; GML, glycerol monolaurate; SG, selenium yeast + glycerol monolaurate. Data are presented as the means ± SEM; * *p* < 0.05 and ** *p* < 0.01.

The integrity of both the placental and intestinal barriers plays a pivotal role in mitigating the risk of LPS translocation from the gut to the fetus. To assess the impact of dietary treatments on sow intestinal permeability, we examined the intestinal barrier indicators DAO and DLA, as depicted in Figure 7B. Our results underscore the importance of these barriers and reveal that both SeY and GML supplementation in the diet led to a remarkable reduction in maternal plasma levels of DAO (*p* < 0.01) and DLA (*p* < 0.01) (Figure 7B), indicative of an enhanced intestinal barrier function. Furthermore, we investigated the effects of different dietary treatments on the maternal placental barrier by evaluating the expression of placental tight junction proteins. The outcomes demonstrate that both SeY and GML significantly increased the expression of ZO-1 (*p* < 0.05) protein and highly significantly improved the expression of claudin-1 (*p* < 0.01) protein (Figure 7A).

### 3.6. Effect of Dietary Addition of SeY and GML on Maternal Placental Development

**Figure 8:**
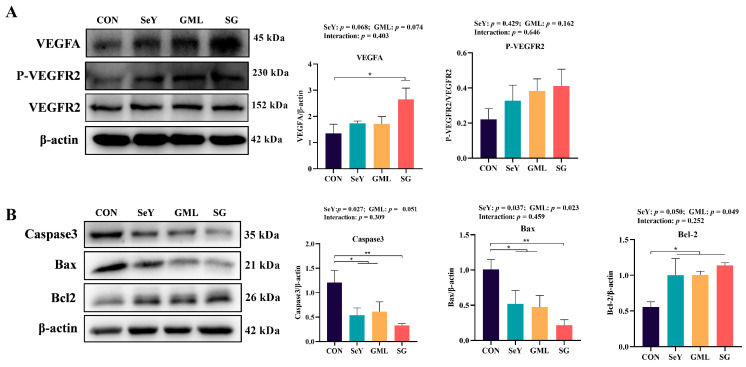
Maternal placental development: (**A**) placental vascular endothelial growth factor signaling pathway (VEGFA and P-VEGFR2/VEGFR2) protein expression; (**B**) placental apoptosis signaling pathway (caspase-3, Bax, and Bcl-2) protein expression. CON, control. SeY, selenium yeast, GML, glycerol monolaurate. SG, selenium yeast + Glycerol monolaurate. Data are presented as the means ± SEM, * *p* < 0.05; ** *p* < 0.01.

Optimal placental development is essential for ensuring fetal growth and wellbeing throughout pregnancy. With the purpose of understanding the effects of SeY and GLM on placental vascular development, we determined the expressions of VEGF and P-VEGFR2, which are closely associated with angiogenesis. We observed that dietary supplementation with both SeY and GML led to the upregulation of VEGF and P-VEGFR2, as illustrated in Figure 8A.

In addition, we delved into the placental apoptosis signaling pathway, as illustrated in Figure 8B. Dietary SeY supplementation resulted in a noteworthy decrease in the expression of caspase-3 (*p* < 0.05) and Bax (*p* < 0.05) proteins, with a noticeable trend toward increased Bcl-2 protein expression (*p* = 0.050). Similarly, the inclusion of GML in the diet significantly reduced the expression of Bax (*p* < 0.05) protein and significantly enhanced the expression of Bcl-2 (*p* < 0.05) protein, with a noticeable trend toward decreased caspase-3 (*p* = 0.051) protein expression.

### 3.7. Effect of Dietary Addition of SeY or GML on Sow Placental Nutrient Transport

**Figure 9:**
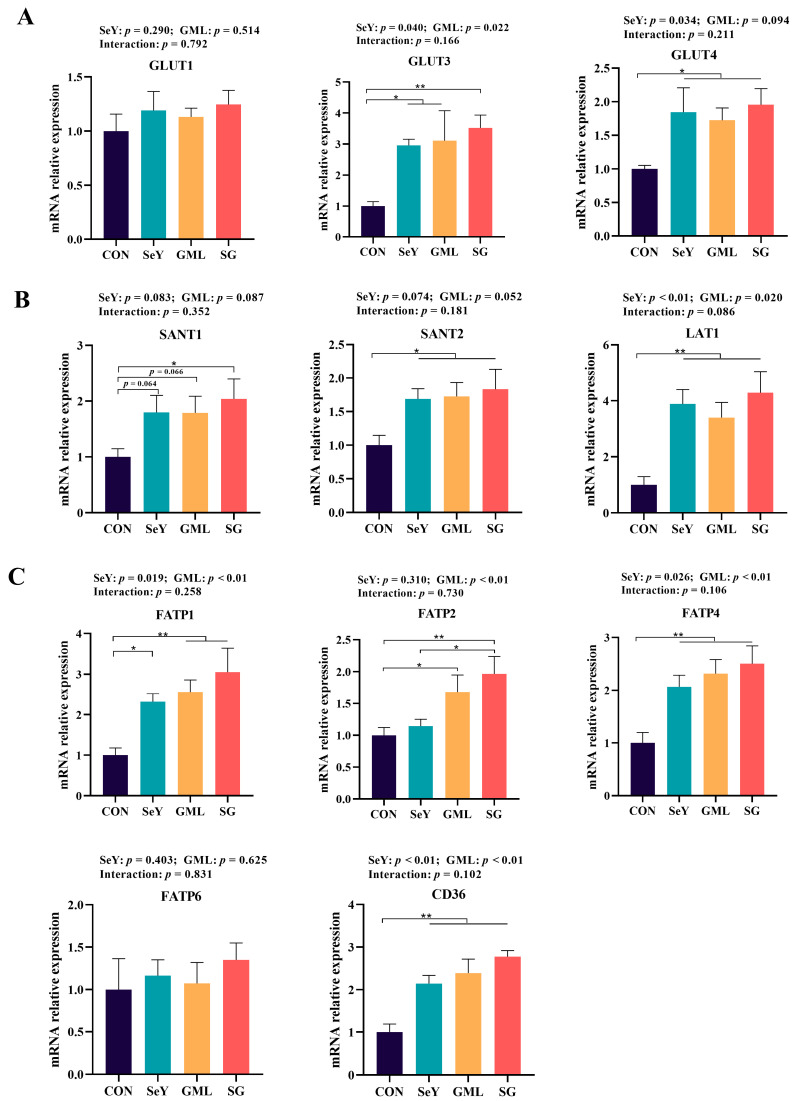
Sow placental nutrient transporter gene expression: (**A**) placental glucose transporter mRNA expression (*GLUT1*, *GLUT3*, and *GLUT4*); (**B**) placental amino acid transporter carrier mRNA expression (*SANT1*, *SANT2*, and *LAT1*); (**C**) placental fatty acid transporter mRNA expression (*FATP1*, *FATP2*, *FATP4*, *FATP6*, and *CD36*). CON, control; SeY, selenium yeast; GML, glycerol monolaurate; SG, selenium yeast + glycerol monolaurate. Data are presented as the means ± SEM; * *p* < 0.05 and ** *p* < 0.01.

Placental nutrient transport is of paramount importance for fetal development, and it relies heavily on specialized amino acid transporters, glucose transporters, and fatty acid transporters located within the placental tissue. Dietary SeY significantly increased the expression of placental glucose transporters *GLUT3* (*p* < 0.05) and *GLUT4* (*p* < 0.05) genes (Figure 9A). It also notably increased the expression of the placental amino acid transporter *LAT1* (*p* < 0.01) gene, with increasing trends for *SANT1* (*p* = 0.083) and *SANT2* (*p* = 0.074) genes (Figure 9B). Moreover, it enhanced the expression of several fatty acid transporters, including the *FATP1* (*p* < 0.05), *FATP4* (*p* < 0.05), and *CD36* (*p* < 0.01) genes (Figure 9C).

The addition of GML to the diet significantly Increased the expression of the placental glucose transporter *GLUT3* (*p* < 0.05) gene and exhibited an upward trend in *GLUT4* (*p* = 0.094) gene expression (Figure 9A). It also significantly enhanced the expression of the placental amino acid transporter *LAT1* (*p* < 0.05) gene, with increasing trends for *SANT1* (*p* = 0.087) and *SANT2* (*p* = 0.052) genes (Figure 9B). Furthermore, it remarkably improved the expression of placental fatty acid transporters, including the *FATP1* (*p* < 0.01), *FATP2* (*p* < 0.01), *FATP4* (*p* < 0.01), and *CD36* (*p* < 0.01) genes (Figure 9C).

### 3.8. Effect of Dietary Addition of SeY and GML on the Gut Microbiota of Sows

**Figure 10:**
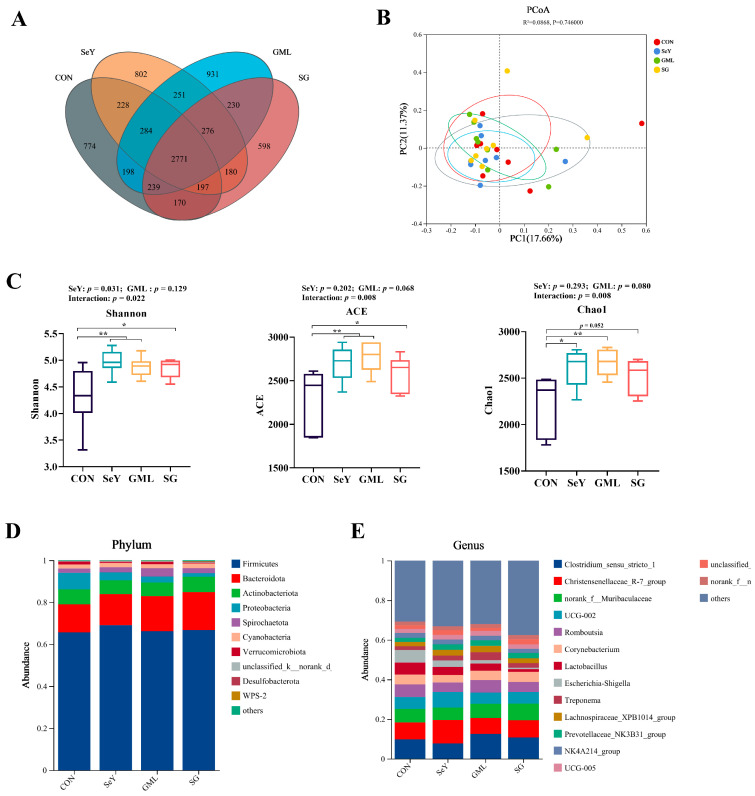
Microbial structure and diversity: (**A**) Venn diagram of the OTUs; (**B**) principal component analysis (pCoA) scores plot; (**C**) Shannon index, ACE index, and Chao1 index of microbiota; (**D**) relative abundance at the phylum level; (**E**) relative abundance at the genus level. CON, control; SeY, selenium yeast; GML, glycerol monolaurate; SG, selenium yeast + glycerol monolaurate. Data are presented as the means ± SEM; * *p* < 0.05 and ** *p* < 0.01.

**Figure 11:**
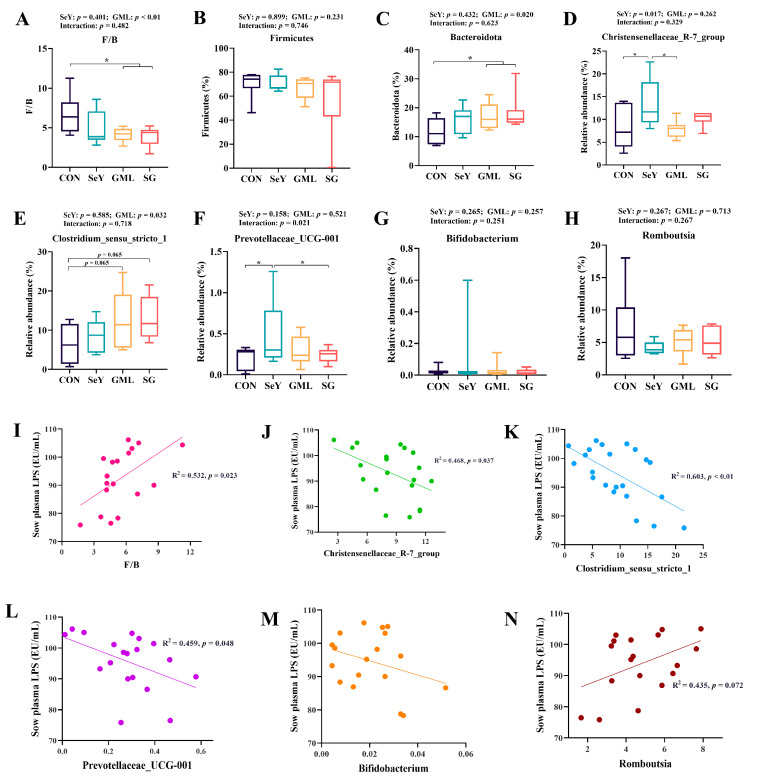
Gut microorganisms for the phylum- and genus-level analysis and regression analysis with sow plasma LPS: (**A**–**H**) microbial phylum- and genus-level analysis; (**I**–**N**) regression analysis of the microbial phylum and genus levels and sow plasma LPS. CON, control; SeY, selenium yeast; GML, glycerol monolaurate; SG, selenium yeast + glycerol monolaurate. Data are presented as the means ± SEM. * *p* < 0.05.

We used 16S rDNA sequencing to analyze the gut microbiota of gestating sows at 110 days of gestation. Our analysis revealed different and shared species among the CON, SeY, GML, and SG groups (Figure 10). Specifically, the CON, SeY, GML, and SG groups contained 774, 802, 931, and 598 unique OTUs, respectively, of which 2771 OTUs were shared among groups (Figure 10A). In addition, we assessed the diversity and structure of the microbial community and observed significant changes induced by different dietary treatments (Figure 10B). In addition to this, there was a trend toward higher ACE (*p* = 0.068) and Chao (*p* = 0.080) indices upon dietary addition of GML (Figure 10B). Interestingly, SeY and GML two factors have an interactive effect on Shannon, ACE, and Chao indices (*p* < 0.05) (Figure 10C). Our analysis also revealed changes in the gut microbiota at the phylum (Figure 10D) and genus levels (Figure 10E) in response to different dietary treatments. Compared to the CON group, the Firmicutes phylum did not show significant changes in the SeY, GML, and SG groups, whereas the Bacteroidota phylum showed an increased tendency (Figure 10D). In order to further determine the changes in the composition of fecal microbiota, further analysis of the genus levels showed that dietary addition of SeY significantly enhanced Christensenellaceae_R-7_ (*p* < 0.05) (Figure 11D); dietary addition of GML highly significantly decreased F/B (*p* < 0.01) (Figure 11A) and significantly increased Bacteroidota (*p* < 0.05) (Figure 11C) and Clostridium_sensus_stricto_1 (*p* < 0.05) (Figure 11E) levels.

Linear regression analysis showed a significant positive correlation (R^2^ = 0.468, *p* < 0.05) between the F/B ratio and sow plasma LPS (Figure 11I). In addition, there was a significant negative correlation between beneficial bacteria Christensenellaceae_R-7_group (R^2^ = 0.532, *p* < 0.05), Clostridium_sensus_stricto_1 (R^2^ = 0.603, *p* <0.01), Prevotellaceae_UCG-001 (R^2^ = 0.459, *p* < 0.05) and sow plasma LPS, while a positive trend was observed with the harmful bacterium Romboutsia (R^2^ = 0.435, *p* = 0.072) (Figure 11J–N).

**Figure 12:**
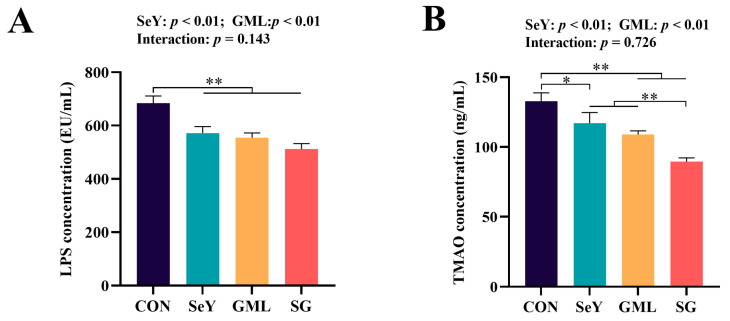
Sow intestinal microbial metabolites. (**A**) Sow fecal LPS content. (**B**) Sow fecal TMAO content. CON, control. SeY, selenium yeast, GML, glycerol monolaurate. SG, selenium yeast + glycerol monolaurate. Dates are presented as means ± SEM, * *p* < 0.05; ** *p* < 0.01.

To further confirm the impact of dietary SeY and GML supplementation on sow gut microbiota, we assessed the levels of LPS and TMAO in the sow plasma. The results demonstrate a significant reduction in their levels (*p* < 0.01) (Figure 12).

## 4. Discussion

This study, for the first time, reveals the pivotal role of gut microbiota-derived endotoxins in mediating the influence of dietary intervention on the transfer of antioxidative capacity from the mother to the fetus through the gut–placenta–fetus axis. These findings provide new insights for understanding the roles of gut microbiota in the beneficial effect of SeY and GLM on adverse pregnancy outcomes during pregnancy.

### 4.1. Intestine-Derived LPS Participates in Mediating the Effects of Selenium and GML

The intricate balance of gut microbiota is increasingly acknowledged as a pivotal determinant in maintaining overall health and mitigating a variety of disorders [24]. Gut dysbiosis has been closely correlated with adverse pregnancy outcomes such as preeclampsia, hypertension, and abortion [25,26,27]. Researchers have noted heightened toxic microbial metabolites in feces, the gut, or the blood of individuals with gut dysbiosis during pregnancy, potentially modifiable through dietary interventions [28,29,30]. Lipopolysaccharides (LPS), large molecules located in the outer membranes of Gram-negative bacteria predominantly inhabiting the gastrointestinal tract, could be released upon bacterial cell death and subsequently enter the bloodstream, possibly translocating to remote organs to induce impairment [31,32]. In the current study, we identified introducing Se and GML to the diet resulted in decreased LPS levels along the maternal–placental–fetal axis and it is negatively related to placental weight. These findings provide initial evidence of the crucial role of LPS in mediating the effects of these two nutritional compounds on placental function. However, it remains uncertain whether LPS is linked to gut microbiota in this context.

### 4.2. Selenium and GML Reshape Sow Gut Microbiota Composition to Mitigate Systemic LPS Translocation from Mother to Fetus

Therefore, we conducted microbial sequencing on fecal samples from sows, with the aim to look the effects of different dietary components on the gut microbiota of sows. We found that adding SeY and GML to the diet increased the beneficial bacteria (Christensenellaceae_R-7_group, Clostridium_sensus_stricto_1, Bacteroidota) abundance and decreacsed the F/B ratio. Christensenellaceae_R-7_group is associated with enhancing intestinal barrier integrity and reducing oxidative stress in the body [33,34]. Clostridium_sensus_stricto_1 has been reported to promote the health of the intestinal barrier by producing short-chain fatty acids (SCFAs), maintain the integrity of the intestinal barrier, and help reduce the entry and penetration of LPS [35,36]. Bacteroidota also play an important role in maintaining the balance of gut microbiota, protecting the intestinal mucosal barrier [37]. In addition, an increased F/B ratio is often associated with gut dysbiosis, such as in overweight and obese individuals and higher F/B ratios in obese patients have been positively correlated with elevated LPS levels [38]. The changed intestinal microbial composition in those sows with dietary treatment potentially led to strengthened gut barrier. To reach the fetus, gut-derived LPS must first traverse the intestinal barrier into the bloodstream, followed by traversal through the placental barrier [39,40]. We observed that SeY and GML supplementation reduced the levels of detrimental gut microbiota-derived metabolites LPS and TMAO in fecal samples. Moreover, all three dietary interventions enhanced the integrity of the intestinal and placental barriers, as evidenced by the downregulation of DLA in the sow plasma and the upregulation of ZO-1 and claudin-1 in the placenta in our study, suggesting a protective role in maintaining the fetal intrauterine environment. These results, ultimately, suggest that these nutritional compounds may reshape gut microbiota composition to mitigate systemic LPS translocation from mother to fetus.

### 4.3. Selenium and GML Enhance the Antioxidative Status of the Placenta and Fetus during Pregnancy

Numerous previous studies across various species, including sow, mice, and cattle, have convincingly demonstrated the significant benefits of dietary supplementation in maintaining the redox balance of pregnant individuals [41,42,43]. Here, our focus shifts to investigating whether and how this maternal benefit could be transmitted to the fetus through the placenta. To counter the challenge posed by ongoing reactive oxygen species (ROS) production, cells have developed an antioxidant defense system comprising enzymatic and nonenzymatic components [44,45]. Our findings reveal notable increases in the placenta and fetal plasma levels of essential enzymatic antioxidants—T-AOC, SOD, GSH, and GSH-PX—in sows subjected to dietary treatment. Furthermore, considering Se’s role as a nonenzymatic element, we measured its concentration in both sow plasma and placenta and consistently observed an increase in the SeY and SG groups. Additionally, we investigated the gene expression of nine selenoproteins, as Se primarily exerts its antioxidant function through this form in the placenta, and noted a similar trend [46,47]. These outcomes collectively suggest that supplementing the diet with Se and GML could enhance the antioxidative status of the placenta and fetus during pregnancy.

### 4.4. Intestine-Derived LPS Modulates the Antioxidative Transmission between the Mother and Fetus

The persistent and intensified oxidative burden could disturb the intricate balance of placental function, potentially initiating an inflammatory cascade characterized by the release of pro-inflammatory cytokines and other immune mediators [48,49]. In the current study, we observed that the supplementation of Se and GML also alleviated inflammation levels in both the placenta and fetus, as indicated by decreased TNF-α, IL-1β, IL-6 and IL-8. This reduction in inflammation corresponds with the observed benefits in redox balance. Additionally, the activation of the Nrf2 signaling pathway, crucial in controlling antioxidative stress, along with the inactivation of the NF-κB signaling pathway, which plays a role in the pro-inflammatory process within the placenta, further confirms the positive impact of Se and GML supplementation on maintaining redox balance. Further data analysis revealed a correlation between these pivotal parameters related to redox balance and the inflammatory response, and the levels of LPS along the maternal–placental–fetal axis. This suggests the significant involvement of LPS in modulating the effects of Se and GML on antioxidative transmission between the mother and fetus.

### 4.5. Placenta Is a Key Functional Site for Antioxidative Transmission between the Mother and Fetus

The placenta, acting as the central interface between the maternal and fetal environments, is pivotal in maintaining an optimal intrauterine environment for fetal growth [50,51]. Throughout pregnancy, the placenta undergoes a dynamic process of angiogenesis—the formation of new blood vessels—which interacts closely with its development [52,53]. This intricate network of blood vessels, encompassing both maternal and fetal components, facilitates essential functions including nutrient uptake, oxygen delivery, and waste elimination, all crucial for supporting fetal development [54,55]. Our study showed a trend of elevated vascular endothelial growth factor (VEGF) signaling pathways with dietary addition of SeY and GML, indicating enhanced angiogenesis. Conversely, apoptosis-related pathways were notably downregulated, evidenced by reduced caspase-3 and Bax expression, as well as increased Bcl-2 expression, highlighting improved placental development. Placental nutrient transporters are specialized proteins located within the placental membrane, facilitate the selective movement of nutrients, from the maternal bloodstream to the developing fetus [56,57]. Their activity and expression profoundly influence fetal nutrient supply [58]. Interestingly, the majority of nutrient transporters involved in amino acids, fatty acids, and glucose exhibited upregulation with Se and GML supplementation, suggesting enhanced nutrient delivery in the placenta. These findings imply that augmented placental development and function could potentially modulate the transmission of antioxidative capacity between mother and offspring.

## 5. Conclusions

Through optimizing gut microbiota and reinforcing intestinal and placental barriers, SeY and GLM supplementation reduces LPS levels across the maternal–placental–fetal axis, contributing to improved placental development and function and, subsequently, alleviating oxidative stress and inflammation within the uterus. These findings provide new insights for understanding the antioxidative capacity transfer between maternal and fetal compartments and revealed in more depth the underlying mechanism of the beneficial effect of SeY and GLM on adverse pregnancy outcomes during gestation.

## Data Availability

The datasets used and/or analyzed during the current study are available from the corresponding author upon reasonable request.

## Supplementary Materials

The following supporting information can be downloaded at: https://www.mdpi.com/article/10.3390/antiox13020141/s1, Table S1: Composition and nutrient levels of basal diets (%); Table S2: Reagent kit information related to chemical analysis; Table S3: Primer sequences used in real-time PCR; Table S4: Information related to primary and secondary antibodies in the Wenstern blot.

## Abbreviations

CON: control; Se: selenium; SeY: selenium yeast; GML glycerol: monolaurate; SG: selenium yeast + glycerol monolaurate; PCoA: principal coordinate analysis; LDA: linear discriminant analysis; ROS: reactive oxygen species; LPS: lipopolysaccharide; DAO: diamine oxidase; DLA: D-lactic acid; TMAO; trimethylamine oxide; T-AOC: total antioxidant capacity; MDA: malondialdehyde; SOD: superoxide dismutase; GSH: glutathione; GSH-PX: glutathione peroxidase; GPX1: glutathione peroxidase 1; GPX3: glutathione peroxidase 3; GPX4: glutathione peroxidase 4; GPX6: glutathione peroxidase 6; SEPHS2: selenophosphate synthetase 2; SELENOP: selenoprotein P; TXNRD1: thioredoxin reductase 1; TXNRD2: thioredoxin reductase 2; TXNRD3: thioredoxin reductase 3; TNF-α: tumor necrosis factor-α; IL-1β: interleukin-1β; IL-6: interleukin-6; IL-8: interleukin-8; IL-12: interleukin-12; IL-18: interleukin-18; TLR4: Toll-like receptor 4; MYD88: myeloiddifferentiationfactor 88; GLUT1: glucose transporter type 1; GLUT3: glucose transporter type 3; GLUT4: glucose transporter type 4; Nrf2: nuclear factor erythroid 2-related factor 2; P-Nrf2: phospho-nuclear factor erythroid 2-related factor 2; Keap1: Kelch-1ike ECH-associated protein l; SOD1: superoxide dismutase 1; SOD2: superoxide dismutase 2; HO-1: heme oxygenase 1; P38: p38 MAPK; P-P38: phospho-p38 MAPK; JNK: C-Jun N-terminal protein kinase; P-JNK: phospho-c-Jun N-terminal protein kinase; ERK: extracellular signal-regulated kinase; P-ERK: phospho-extracellular signal-regulated kinase; NF-Κb: nuclear factor kappa-B; P-NF-κB: phospho-nuclear factor kappa-B; ZO-1: zonula occludens protein 1; VEGF-A: vascular endothelial growth factor A; VEGFR-2: Vascular endothelial growth factor receptor 2; P-VEGFR-2: phospho-vascular endothelial growth factor receptor 2; caspase-3: cysteinyl aspartate specific proteinase 3; Bax: BCL2-associated X protein; Bcl-2: apoptosis regulator Bcl-2.
