# Supplementary material for: Enhanced Antioxidative Capacity Transfer between Sow and Fetus via the Gut–Placenta Axis with Dietary Selenium Yeast and Glycerol Monolaurate Supplementation during Pregnancy
Source: Antioxidants (Basel). 2024 Jan 23;13(2):141. doi: 10.3390/antiox13020141 (PMC10886224; doi:10.3390/antiox13020141)
Supplement: Supplementary file 1 [file antioxidants-13-00141-s001.zip › antioxidants-2774960-supplementary.pdf]

Tables

Table S1 Composition and nutrient levels of basal diets (%).

Item	Late Gestation
Ingredients	
Corn	47.90
Barley	30.00
Bran	5.00
Soybean meal (43%)	12.10
Fish meal	1.00
Limestone	1.00
$CaHPO_4$	1.20
Salt	0.50
Lys	0.15
DL-Met	0.15
Premix [1]	1.00
Total	100
DE, MJ/kg	3150.00
CP	13.50
EE	2.50
CF	3.24
Ash	5.18
Ca	0.95
TP	0.60
AP	0.35
TLys	0.70

[1] The premix provided the following per kg of diets: VA 10,000 IU, VD_3 2100 IU, VE 45 IU, VK_3 2.0 mg, thiamine 3.0 mg, riboflavin 5.0 mg, VB_6 1.8 mg, VB_{12} 0.03 mg, choline chloride 1000 mg, nicotinic acid 25.0 mg, pantothenic acid 15.0 mg, biotin 0.08 mg, folic acid 1.0 mg, Mn 20.0mg, Zn 80.0 mg, Fe 80 mg, Cu 6.0 mg, I 0.10 mg, Se 0.30 mg.

Table S2 Reagent kit information related to chemical analysis.

Kits	Product name	Code No.	Company
LPS	Porcine LPS kit	YJ790293	Yuanjie company(Shanghai,China)
DAO	Porcine Diamine Oxidase (DAO) ELISA Kit	YJ002413	Yuanjie company(Shanghai,China)
DLA	Porcine D-lactic acid(DLA)	YJ791023	Yuanjie company(Shanghai,China)
TMAO	Porcine TMAO kit	YJ021122	Yuanjie company(Shanghai,China)
TNF-a	Porcine Tumor Necrosis Factor-α (TNF-α) ELISA Kit	YJ002360	Yuanjie company(Shanghai,China)
IL-1β	Porcine Interleukin IL-1B ELISA Kit	YJ022366	Yuanjie company(Shanghai,China)
IL-6	Porcine Interleukin IL-6 ELISA Kit	YJ663251	Yuanjie company(Shanghai,China)
Il-8	Porcine Interleukin 8 (IL-8/CXCL8) ELISA Kit	YJ002320	Yuanjie company(Shanghai,China)
IL-18	Porcine Interleukin 18 (IL-18) ELISA Kit	YJ002299	Yuanjie company(Shanghai,China)
T-AOC	Total antioxidant capacity assay kit(ABTS method)	A-015-2-1	Nanjing JianCheng Bioengineering Institute
T-SOD	(Hydroxylamine method) Total Superoxide Dismutase(T-SOD) assay kit	A001-1-2	Nanjing JianCheng Bioengineering Institute
GSH-Px	Glutathione peroxidase (GSH-PX) assay kit	A005-1-2	Nanjing JianCheng Bioengineering Institute
GSH	Reduced glutathione (GSH) assay kit	A006-2-1	Nanjing JianCheng Bioengineering Institute
MDA	Malondialdehyde(MDA)assay kit(TBA method)	A003-1-2	Nanjing JianCheng Bioengineering Institute
Lysis buffer		EZB-RN001	EZBioscience,USA
Tissue RNA Purification		EZB-RN001	EZBioscience,USA
Colour Reverse Transcription Kit(with gDNA Remover)		A0010CGQ	EZBioscience,USA
Colour SYBR Green qPCR Master Mix kit		A0012-R2	EZBioscience,USA
RIPA Lysis Buffer P0013B Beyotime		P0013B	Beyotime, Shanghai, China
BLOT-QuickBlocker		C006011	Sangon, Shanghai,China
BCA Protein Assay Kit		P0012	Beyotime, Shanghai, China
ECL Plus chemiluminescence detection kit		P1010-250	Applygen Technologies Inc., Beijing, China

1

Table S3 Primer sequences used in real-time PCR.

Gene Name	Gene Accession		Primer Sequences (5′→3′)
GPX1	AF532927	Forward	F: GATGCCACTGCCCTCATGA
		Reverse	R: TCGAAGTTCCATGCGATGTC
GPX3	AY368622	Forward	F: TGCACTGCAGGAAGAGTTTGAA
		Reverse	R: CCGGTTCCTGTTTTCCAAATT
GPX4	NM_214407	Forward	F: TGAGGCAAGACGGAGGTAAACT
		Reverse	R: TCCGTAAACCACACTCAGCATATC
GPX6	NM_001137607	Forward	F: GAGCTGAAGCCTTTTGGTGTAGTT
		Reverse	R: CTTTGCTGGTTCTTGTTTTCCA
SEPHS2	EF033624	Forward	F: TGGCTTGATGCACACGTTTAA
		Reverse	R: TGCGAGTGTCCCAGAATGC
SELENOP	EF113596	Forward	F: AACCAGAAGCGCCAGACACT
		Reverse	R: TGCTGGCATATCTCAGTTCTCAGA
TXNRD1	AF537300	Forward	F: GATTTAACAAGCGGGTCATGGT
		Reverse	R: CAACCTACATTCACACACGTTCCT
TXNRD2	GU181287	Forward	F: TCTTGAAAGGCGGAAAAGAGAT
		Reverse	R: TCGGTCGCCCTCCAGTAG
TXNRD3	BX918808	Forward	F: GTGCCCTACGTTTATGCTGTTG
		Reverse	R: TCCGAGCCACCAGCTTTG
TNF-α	NM_214022.1	Forward	CCACCAACGTTTTCCTCACT
		Reverse	TAGTCGGGCAGGTTGATCTC
IL-1β	NM_214055.1	Forward	TCTGCCCTGTACCCCAACTG
		Reverse	CCAGGAAGACGGGCTTTTG
IL-6	AF518322.1	Forward	TGGCTACTGCCTTCCCTACC
		Reverse	AGAGCCTGCATCAGCTCAGT
IL-8	NM 213867	Forward	AAATACGCATTCCACACCTTTCCAC
		Reverse	TGCTGTTGTTGTTGCTTCTCAGTTC
IL-12	NC_010458.4	Forward	CAACCCTGTGCCTTAGCAGT
		Reverse	AGAGCCTGCATCAGCTCAGT
IL-18	NP_999162.1	Forward	CAAGCCGTGTTTGAGGATATGCC
		Reverse	GGTTACTGCCAGACCTCTAGTGAG
TLR4	NM_001113039.1	Forward	CAACCCTGTGCCTTAGCAGT
		Reverse	AGAGCCTGCATCAGCTCAGT
MYD88	NM_001099923	Forward	CGCATGGTGGTGGTTGTT
		Reverse	GCCTTCTTCATCGCCTTGTATTT
GLUT1	NW_003610563.1	Forward	GATGAAGGAGGAGTGCCG
		Reverse	CAGCACCACGGCGATGAGGAT
GLUT3	XM_021092391.1	Forward	F: TCTCCATCATGCTCCAGCTCTCC
		Reverse	R: AATAGTGGCATAGATTGGCTCCTGAAC
GLUT4	NM_0011288433	Forward	TATGTTGCGGATGCTATGGG
		Reverse	CTCGGGTTTCAGGCACTTTT
SNAT1	XM_003355629	Forward	F: AAGAACCTGGGCTATCTCGG
		Reverse	R: TGTTGCGTTAGGACTCGTTG
SNAT2	NM_018976	Forward	F: GTTACCTTTGGTGATCCAGGC
		Reverse	R: ACCAATGACACCAGCAGAACC
LAT1	NM_003486	Forward	F: GCCCATTGTCACCATCATC
		Reverse	R: GAGCCCACAAAGAAAAGC
FATP1	NM_001083931.1	Forward	GGCAACAGACGTGATCTATGAC
		Reverse	AGCGGCTGGCTGAAAACT

FATP2	JX092264.1	Forward	TCTAACACGGATGGGGTCG
		Reverse	AGGGCAGGAGTGGAAAAGT
FATP4	XM_003353676.1	Forward	AGCCGCATCCTGTCCTTT
		Reverse	GACATCCTTGGCGATCTTTT
FATP6	XP_020940394.1	Forward	ACGCAGCCACCATGTTGTCTC
		Reverse	GCCCACAGGACAGCAGAACC
CD36	DQ192230.1	Forward	GGACTCATTGCTGGTGCTGT
		Reverse	GTCTGTAAACTTCCGTGCCTGT
β-actin	XM_021086047.1	Forward	GATCTGGCACCACACCTTCTACAAC
		Reverse	TCATCTTCTCACGGTTGGCTTTGG

Table S4 Information related to primary and secondary antibodies in the Wenstern blot.

Protein	Product name	Code No.	Company
Nrf2	NRF2, NFE2L2 Polyclonal antibody	16396-1-AP	Proteintech
P-Nrf2	Phospho-Nrf2 (Ser40) Rabbit pAb	381559	Zenbio
Keap1	KEAP1 Polyclonal antibody	10503-2-AP	Proteintech
SOD1	SOD1 Polyclonal antibody	10269-1-AP	Proteintech
SOD2	SOD2 Polyclonal antibody	24127-1-AP	Proteintech
HO-1	HO-1/HMOX1 Polyclonal antibody	10701-1-AP	Proteintech
P38	p38 MAPK Monoclonal antibody	66234-1-Ig	Proteintech
P-P38	Phospho-p38 MAPK (Thr180/Tyr182) (3D7) Rabbit mAb (Biotinylated) #4092	4092S	Cellsignaling
JNK	SAPK/JNK Antibody #9252	9252S	Cellsignaling
P-JNK	Phospho-SAPK/JNK (Thr183/Tyr185) (81E11) Rabbit mAb #4668	4668S	Cellsignaling
ERK	p44/42 MAPK (Erk1/2) Antibody #9102	9102S	Cellsignaling
P-ERK	Phospho-p44/42 MAPK (Erk1/2) (Thr202/Tyr204) Antibody #9101	9101S	Cellsignaling
NF-κB	NF-κB p65 Polyclonal antibody	10745-1-AP	Proteintech
P-NF-κB	Phospho-NF-κB p65 (Ser536) (93H1) Rabbit mAb #3033	3033S	Cellsignaling
ZO-1	ZO-1 Polyclonal antibody	21773-1-AP	Proteintech
Occlaudin	Occludin Polyclonal antibody	27260-1-AP	Proteintech
Claudin-1	Claudin 1 Polyclonal antibody	13050-1-AP	Proteintech
VEGF-A	VEGFA Polyclonal antibody	19003-1-AP	Proteintech
VEGFR-2	VEGFR2 Polyclonal antibody	26415-1-AP	Proteintech
P-VEGFR-2	Phospho-VEGF Receptor 2 (Tyr1175) (19A10) Rabbit mAb #2478	2478S	Cellsignaling
Caspase3	Caspase 3/p17/p19 Polyclonal antibody	19677-1-AP	Proteintech
Bax	BAX Polyclonal antibody	50599-2-Ig	Proteintech
Bcl-2	Bcl2 Polyclonal antibody	26593-1-AP	Proteintech
secondary antibody	Goat Anti-Rabbit IgG H&L	511203	Zenbio
secondary antibody	Goat Anti-Mouse IgG H&L	511103	Zenbio
